# Sialic Acid Metabolic Engineering: A Potential Strategy for the Neuroblastoma Therapy

**DOI:** 10.1371/journal.pone.0105403

**Published:** 2014-08-22

**Authors:** Vinayaga S. Gnanapragassam, Kaya Bork, Christina E. Galuska, Sebastian P. Galuska, Dagobert Glanz, Manimozhi Nagasundaram, Matthias Bache, Dirk Vordermark, Guido Kohla, Christoph Kannicht, Roland Schauer, Rüdiger Horstkorte

**Affiliations:** 1 Institute for Physiological Chemistry, Martin-Luther-University Halle-Wittenberg, Halle (Saale), Germany; 2 Institute of Biochemistry, Faculty of Medicine, University of Giessen, Giessen, Germany; 3 Clinic of Radiotherapy, University Hospital Halle, Halle (Saale), Germany; 4 Octapharma R&D, Molecular Biochemistry, Berlin, Germany; 5 Institute of Biochemistry, University of Kiel, Kiel, Germany; National Cancer Institute at Frederick, United States of America

## Abstract

**Background:**

Sialic acids (Sia) represent negative-charged terminal sugars on most glycoproteins and glycolipids on the cell surface of vertebrates. Aberrant expression of tumor associated sialylated carbohydrate epitopes significantly increases during onset of cancer. Since Sia contribute towards cell migration ( =  metastasis) and to chemo- and radiation resistance. Modulation of cellular Sia concentration and composition poses a challenge especially for neuroblastoma therapy, due to the high heterogeneity and therapeutic resistance of these cells. Here we propose that Metabolic Sia Engineering (MSE) is an effective strategy to reduce neuroblastoma progression and metastasis.

**Methods:**

Human neuroblastoma SH-SY5Y cells were treated with synthetic Sia precursors N-propanoyl mannosamine (ManNProp) or N-pentanoyl mannosamine (ManNPent). Total and Polysialic acids (PolySia) were investigated by high performance liquid chromatography. Cell surface polySia were examined by flow-cytometry. Sia precursors treated cells were examined for the migration, invasion and sensitivity towards anticancer drugs and radiation treatment.

**Results:**

Treatment of SH-SY5Y cells with ManNProp or ManNPent (referred as MSE) reduced their cell surface sialylation significantly. We found complete absence of polysialylation after treatment of SH-SY5Y cells with ManNPent. Loss of polysialylation results in a reduction of migration and invasion ability of these cells. Furthermore, radiation of Sia-engineered cells completely abolished their migration. In addition, MSE increases the cytotoxicity of anti-cancer drugs, such as 5-fluorouracil or cisplatin.

**Conclusions:**

Metabolic Sia Engineering (MSE) of neuroblastoma cells using modified Sia precursors reduces their sialylation, metastatic potential and increases their sensitivity towards radiation or chemotherapeutics. Therefore, MSE may serve as an effective method to treat neuroblastoma.

## Introduction

Sialic acids (Sia) are 9-carbon acidic monosaccharides located at the terminal position of the *N*- or *O*-glycans on glycoproteins or glycolipids. They exist as mono, di, oligo and polymeric forms and fine-tune the function of the associated proteins and lipids [1]-[4]. Most cell surface glycans are highly sialylated and often involved in cell-cell and/or cell-extracellular matrix interaction. This involvement is mediated through I. their negative charge and II. by their interaction with specific Sia-binding proteins (Sia-lectins). Through this interaction Sia play an important role in various biological processes such as growth, development, immunology, pathology and host-pathogen interactions [2]–[5].

Aberrant expression of sialylation has been observed as a characteristic feature for a variety of cancers [6], [7], which enables cancer cells to escape from the immune surveillance and support these cells for increased migration and metastatic rates. Ligands for Sia-lectins, which are generated by sialyltransferases bind to Sia-lectins, such as selectins at the metastatic site and expand the cancer cell population. In addition, Sia also protect cancer cells from chemo- and radiation-therapy [1], [2], [8], [9], [10], [11]. Tumor cells of neural origin often express the neural cell adhesion molecule 1 (NCAM1). This is known to be posttranslationally modified at the IgG domain V of N-glycans by polySia [12]-[14]. PolySia have a unique structure of repeated units and can reach chain lengths of more than 50 sialic acid residues in mammals [14], which is primarily expressed on neural cell adhesion molecule 1 (NCAM 1). Polysialylation of NCAM is generated by specific polysialyltransferases (ST8Sia2 and ST8Sia4) [12], [13], [15].

The biosynthesis of Sia starts in the cytosol. The physiological precursor of all Sia is *N*-acetylmannosamine (ManNAc). In a series of studies we provided evidence that the cellular Sia content can be metabolically engineered simply by feeding cells with synthetic *N*-acyl-modified D-mannosamines such as ManNProp or ManNPent. These synthetic Sia-precursors are taken up by the cells and are efficiently metabolized by the cellular sialylation machinery to the respective Sia (Neu5Prop in the case of ManNProp or Neu5Pent in the case of ManNPent) [18] (Figure S1).

Neuroblastoma is one of the most frequent pediatric cancers, affecting children worldwide. The heterogeneity and complexity arose from various mutations. v-myc avian myelocytomatosis viral oncogene neuroblastoma derived homolog (MYCN) or anaplastic lymphoma receptor kinase (ALK), amplification and other genetic factors decide the aggressiveness and recurrence nature of neuroblastoma [16], [17]. Since neuroblastoma has restricted targets for the tumor specific immunotherapy, it is a challenge to develop a novel strategy to treat aggressive neuroblastoma. Here we focused on targeting hyper-sialylation through MSE using neuroblastoma cells and modified Sia-precursors. We found that depending on the MSE strategy, sialylation and polysialylation of neuroblastoma cells are reduced and that as a consequence migration and invasion are decreased and the sensitivity towards anticancer drugs, such as 5-fluorouracil or cisplatin and radiation, is increased.

## Materials and Methods

### Cell culture

#### Metabolic Sia engineering on SHSY5Y cells

SHSY5Y cells were obtained from DSMZ-German Collection of Microorganisms and Cell Cultures (DSMZ, Braunschweig, Germany), and cultured in DMEM (Dulbecco's Modified Eagle's Medium) high glucose medium containing 10% fetal calf serum, 100 units penicillin, 100 µg streptomycin, 2 mM L-glutamine (PAA, Germany and Invitrogen USA). Prior to MSE cells were seeded at defined numbers based on the size of the flask, and cultured for 24 h. The medium contains ManNAc, ManNProp or ManNPent at 10 mM concentration and was replaced for every 24 h, and incubation was continued for 3 days. Cells cultured without Sia precursor served as a control. Sia precursors were synthesized in our laboratory as previously described [18].

5-Fluorouracil and cisplatin were added separately at various concentrations to the cells previously engineered with modified Sia precursor. For the realtime analysis the cells were prepared as described for the migration assay with some modification. Cells (10,000/well) were added and allowed to grow for 18 h until the cell index reached the log phase. Fresh medium containing ManNProp or ManNPent and the anticancer drugs were replaced every 24 h, and the cultivation was continued for 72 h. Impedance (measurement of opposition by a circuit presenting to a current on application of voltage) was measured for each 15 min until 72 h.

### Flow cytometry

0.05×10^6^ Cells were seeded in 24 well plates and engineered with Sia precursors for 3 days. Cells were washed with ice-cold PBS and dislodged with PBS/EDTA buffer. Dissociated cells were pelleted by centrifugation at 0.4×g for 5 min and washed twice with 1 ml of PBS. 100 µl of RPMI-1640 (Roswell Park Memorial Institute) contains 5% FCS was added and gently mixed to disperse the cell pellet. 1∶100 dilution of anti-polySia (735) primary antibody (a kind gift from Prof. Dr. Rita Gerardy-Schahn) was added and the mixture incubated on ice for 60 min, followed by 2 times washing of the cells with 1 ml ice-cold PBS. 100 µl of RPMI containing 5% FCS was added followed by the addition of anti-mouse secondary antibody (Dianova GmbH, Germany) conjugated with fluorophore Dylight 488 (1∶100) and incubated on ice for 45 min, followed by washing the cells twice with 1 ml of ice-cold PBS. 600 µl of fresh ice-cold PBS was added and measured in the BD calibur flow cytometer.

### High performance liquid chromatography

**Sample preparation.** Sia engineered cells and control cells were disassociated and pelleted by centrifugation at 0.4×g for 5 min. For the total Sia analysis cells were homogenized and hydrolyzed with 5 ml of 2 M formic acid at 80°C for 4 h in a water bath. Samples were cooled on ice for 10 min, followed by anion-exchange purification by gravity flow, using prepacked 2 ml BioRad 2-X8 anion-exchange resin columns Cl^-^. Elution was performed with 10 ml of 1 M formic acid followed by column wash with ultrapure water. Both the eluate and the column wash was mixed and lyophilized, as described in the literature [19]. Samples were derivatized with 50 µl of DMB reagent and 10 µl of 2 M acetic acid, by incubating at 56°C for 60 min in the dark.

### Analysis of total sialic acids

DMB derivatized samples were serially diluted and 10 µl was injected into the reversed phase Gemini-NX C18 110 Å (particle size 3 µm, 4.6×150 mm) column attached to the Dionex Ultimate 3000 HPLC system. Chromatography was performed by isocratic elution (1 ml/min) using the solvent methanol, acetonitrile and water mixed to a ratio of 6/8/86 (v/v/v) by the gradient pump of the HPLC system. The elution was monitored by a fluorescence detector at 448 nm emission and 373 nm excitation for 40 min [20].

### Sample preparation and HPLC analysis of polysialic acid

To analyze the chain-length pattern of polySia a DMB–HPLC method was used as described previously [21 22]. To this end 80% of the homogenized cell samples were dissolved in 240 µl DMB reaction buffer (9 mM sodium hydrosulfite, 1 M β-mercaptoethanol, 20 mM trifluoroacetic acid (TFA) and 2.7 mM DMB (Dojindo, Kumamoto, Japan), were incubated for 24 h at 11°C. The reaction was stopped by adding 60 µl of 1 M NaOH and released polySia chains were separated by HPLC on a DNAPac PA-100 column (Dionex); DMB labeled polySia chains were detected with a fluorescence detector set to 372 nm for excitation and 456 nm for emission to monitor the HPLC run. MilliQ water (M1) and 4 M ammonium acetate (M2) were used as mobile phases at a flow rate of 1 ml/min. Elution was performed by the following gradient: T_0_ min = 0% M2; T_15 min_ = 8% M2; T_20 min_ = 11% M2; T_35 min_ = 14% M2; T_55 min_ = 16% M2; T_100 min_ = 20% M2 and T_125 min_ = 24% M2. The column was washed with 100% M2 for 10 min.

### Functional assays

#### Migration assay

The migration assay was performed in a real-time cell analyzer (RTCA, OLS xCELLigence, Bremen, Germany). Cells were Sia engineered with sialic acid precursors for 24 h, washed twice with 10 ml of PBS and dislodged with PBS/EDTA buffer, and washed once with serum free medium. Cells (50,000/well) were added to the upper chamber of the 16 well CIM plate (OLS xCELLigence, Bremen, Germany). The lower chamber had been previously filled with 160 µl of complete medium. After the cells were settled in the upper plate (kept in the cell culture hood for 30 min), the CIM plate was placed in the station. Migration of the cells was monitored by measuring the impedance for every 15 min up to 24 h.

#### Invasion assay

ECM protein was coated in the upper chamber of the CIM plate and allowed to bind for 4 h at 37°C. Cells were prepared as described in the migration assay, and cells (50,000/well) were seeded in the upper chamber. The lower chamber had been previously filled with serum containing medium. Then the CIM plate was kept aside in the cell culture hood for 30 min to settle the cells. The impedance was measured every 15 min for 24 h.

#### 3-[4,5-Dimethylthiazol-2-yl]-2,5 diphenyl tetrazolium bromide (MTT) based cell viability assay

Cells (10,000/well) were seeded in the 96 well plates and cultured for 18 h. 5-FU was added at various concentrations. The cells were cultured in the presence or absence of 10 mM of ManNProp or ManNPent replaced with fresh medium for every 24 h. After 48 h media were aspirated and replaced with 100 µl of fresh medium, to that 20 µl of 5 mg/ml of MTT reagent was added and incubated for 4 h at 37°C. Then media were aspirated and 150 µl of DMSO was added to each well. The plates were kept in a plate shaker for 20 min followed by measuring the absorbance at 560 nm in the ELISA plate reader (Thermo Scientific Multiskan EX Langenselbold, Germany).

#### Radiation treatment on sialic acid engineered cells

Cells were seeded in the T25 flask and allowed to grow for 18 h, and cells were treated with 10 mM ManNProp and ManNPent for 24 h. Irradiation was delivered by a linear accelerator at a total of 6 Gray.The cells were kept in the incubator for 30 min, prior to the functional assay under hypoxic condition, by using cobalt chloride at 100 µM concentration.

#### Statistical analysis

All experiments were performed 3 to 4 times and statistical analysis was performed by Student's *t* test (unequal variances, two-tailed). P<0.05 considered significant.

## Results

### Sia precursors interfered with polysialylation in neuroblastoma cells

**Figure 1:**
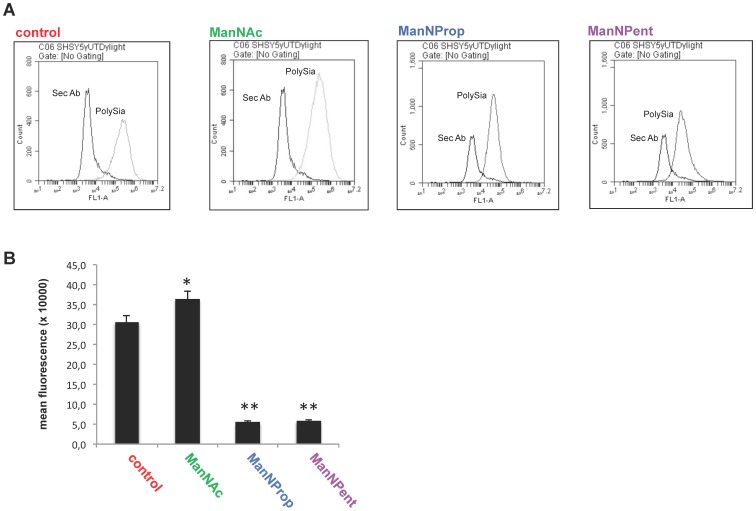
Flow cytometry analysis of cell surface polySia. SHSY5Y cells were cultured in the presence or absence of 10 mM ManNAc, ManNProp or ManNPent for 72 h. Cells were dispersed and analyzed for the polySia status by flow cytometry. The mean fluorescence was recorded and remained the same for the 3 independent experiments. (A, B) ManNProp and ManNPent treatment reduced the polysialic acid quantity significantly (**p<0.0001) whereas ManNAc increased polySia (*P<0.009) in comparison to the untreated control.

**Figure 2:**
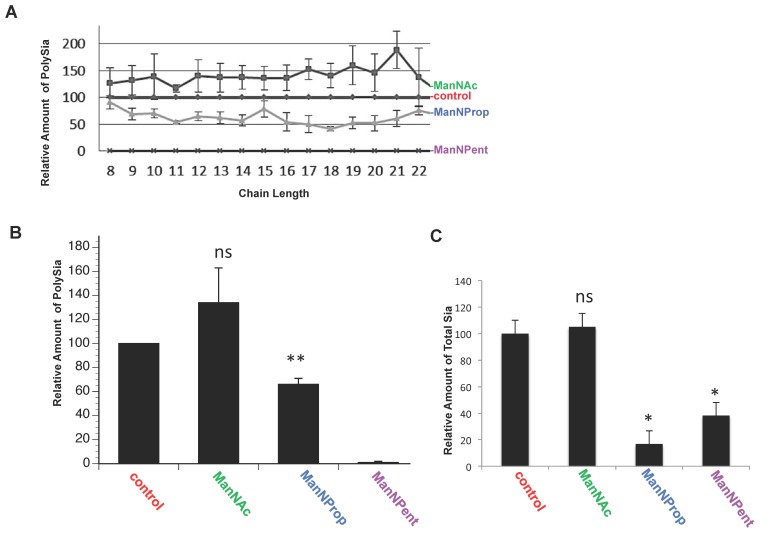
Chromatographic polySia and total Sia analysis of SHSY5-cells cultured with Sia precursors. A. PolySia chains were directly released from cell homogenates and labeled with DMB in a one-pot reaction. Resulting fluorescently tagged sialic acid polymers were separated on an anion-exchange column according to the polySia chain length. Respective chain length of polySia residues is given for selected peaks. B. To analyze possible alterations in the relative proportions of individual polySia chains, Peak areas corresponding to polySia chains ≥DP 8 were calculated to obtain the individual chain length distribution of polySia in lysates from SHSY5Y cells treated with ManNAc, ManNProp and ManNPent as well as untreated cells. Data are means ±S.D. of four independent experiments. Values obtained for untreated cells were set to 100%. The statistical evaluation was performed by Student's t test (unequal variances, two-tailed). Significance levels are indicated by *, p<0.05, **, p<0.005, ns: not significant. C. SHSY5Y cells were cultured in the presence or absence of 10 mM ManNAc, ManNProp or ManNPent. Sia were released with 2 M formic acid and purified by anion-exchange chromatography. Purified sialic acids were tagged with DMB and analyzed by reversed phase HPLC. Quantification of Sia exhibited that ManNProp treatment led to significant reduction of Sia up to 84% and similarly ManNPent treatment reduced Sia to 60% (*, P<0.01, P<0.03). ManNAc treatment increases the sialic acid concentration 5% more than the untreated control but not significantly (ns: not significant) (A, B).

In a first series of experiments we quantified the polySia expression of SH-SY5Y cells in the presence or absence of natural (ManNAc) and modified (ManNProp and ManNPent) Sia precursors by flow cytometry. SH-SY5Y cells express high levels of polySia (Fig.1A control) and application of the physiological Sia precursor ManNAc led to an increase of polySia expression by approximately 15% (Fig.1A ManNAc). In contrast, metabolic Sia engineering through application of non-natural sialic acid precursors led to reduced cell surface polysialylation as indicated by the reduced mean fluorescence compared to the untreated control. Treatment with ManNProp and ManNPent reduced cell surface polysialylation by nearly 90% (Fig.1A ManNProp, ManNPent). Figure 1B summarizes the data on polySia shown before. These experiments have proved for the first time that cell surface polySia expression on the neuroblastoma cells can be regulated by the application of modified Sia precursors. Since artificial sialic acids may influence the antibody binding during flow cytometry, polysialylation of SH-SY5Y cells was additionally characterized via HPLC after application of the physiological or non-natural Sia precursors (Fig.2 A–B). Application of ManNAc to the SH-SY5Y cells led to an increase in total polySia by 35%. As expected ManNProp reduced the synthesis of polySia chain up to 60% in comparison to untreated cells. This effect was much more pronounced in the case of ManNPent leading to a complete loss of polySia. Treatment with natural as well as modified Sia precursors had no significant cytotoxicity by themselves towards the treated cells (data not shown).

### Sia precursors interfered with sialylation in general

SH-SY5Y cells were cultured in the presence or absence of natural as well as non-natural Sia precursors. Sia were released by acid hydrolysis and purified free sialic acids were quantified by reversed phase HPLC (Fig.2 C). We found only a slight and not significant increase of total Sia after application of the physiological Sia precursor ManNAc, but ManNProp and ManNPent decreased the Sia quantity significantly. Sia content was reduced in the presence of ManNProp by 83% and in the presence of ManNPent by 62%. Independent investigation by HPLC-ESI-MS/MS reconfirmed these data. Interestingly, ManNProp treatment showed more reduction of total natural Sia in comparison to ManNPent treatment and increased formation of corresponding non-natural Sia (data not shown).

### Metabolic Sia engineering with ManNProp or ManNPent leads to reduced migration and invasion

**Figure 3:**
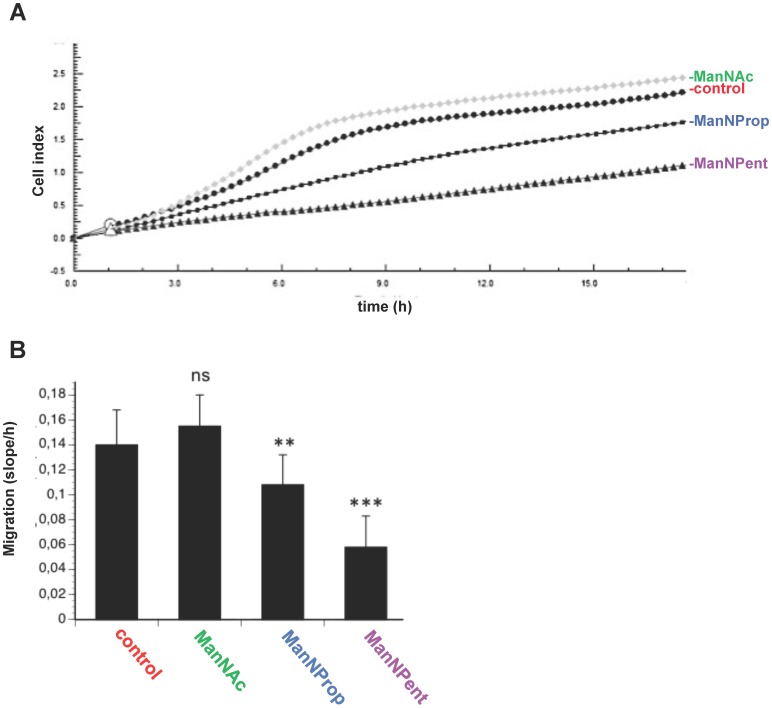
Migration assay. SHSY5Y cells were cultured in the presence of 10 mM ManNAc, ManNProp or ManNPent. Cells were seeded in the CIM plate, using complete medium as a chemo -attractant. A. Cell migration was measured by impedance and read out as cell index. B. Changes in the 1 h of measurement are represented as bars along with standard deviation. ManNProp and ManNPent inhibit the migration with higher significance (P<0.0001, P<0.0001), ns: not significant.

**Figure 4:**
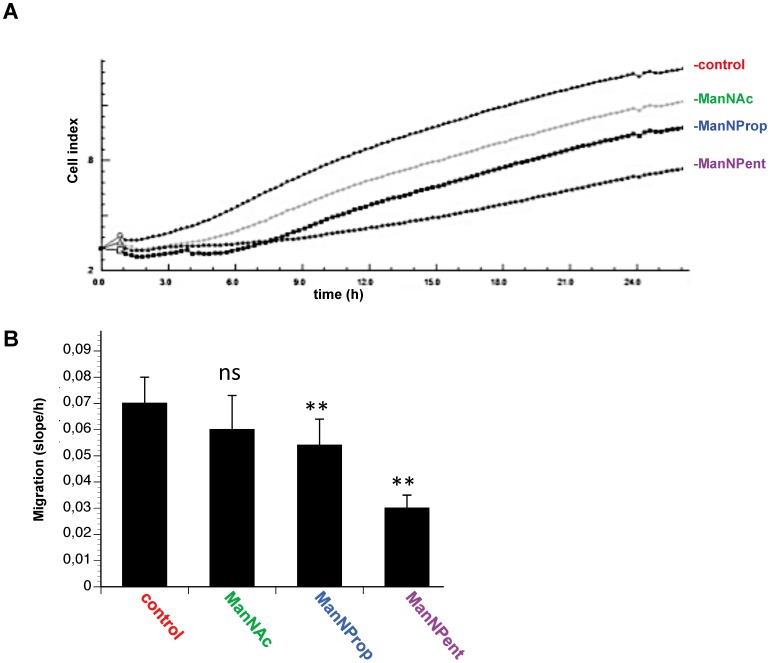
Invasion assay. CIM plate upper chamber was coated with ECM protein for 4 h and the SHSY5Y cells were cultured in the presence or absence of 10 mM ManNAc, ManNProp or ManNPent followed by (A) monitoring the impedance for 24 h. (B) Non-natural Sia precursor treated cells significantly decreased the invasion ability (** p<0.0001) compared with untreated and ManNAc treated cells (**P<0.0002), ns: not significant.

Since sialylation is known to be involved in migration of cells, we analyzed migration of SH-SY5Y cells, which were cultured in the presence or absence of Sia precursors under real-time conditions (Fig.3 A, B). In this type of assay we used serum-containing medium as attractant. Again, ManNAc-treated cells displayed a slightly but not significantly increased migration. However, cells engineered with the Sia precursor ManNProp displayed a significant reduction of approximately 25% in migration and performing the experiments with ManNPent resulted in a nearly 60% decreased migration rate. We then analyzed the invasion of SH-SY5Y cells using real-time device coated with an artificial extra cellular matrix mixture. We found that untreated control cells were very effective in their invasion ability and that application of the physiological Sia precursor ManNAc slightly reduced invasion ability of SH-SY5Y cells (Fig.4 A, B). However, metabolic engineering using ManNProp or ManNPent reduced the invasion of the SH-SY5Y cells significantly (Fig.4 A, B).

### Metabolic Sia engineering with ManNProp or ManNPent leads to an increased sensitivity towards chemotherapeutics and radiation treatment

**Figure 5:**
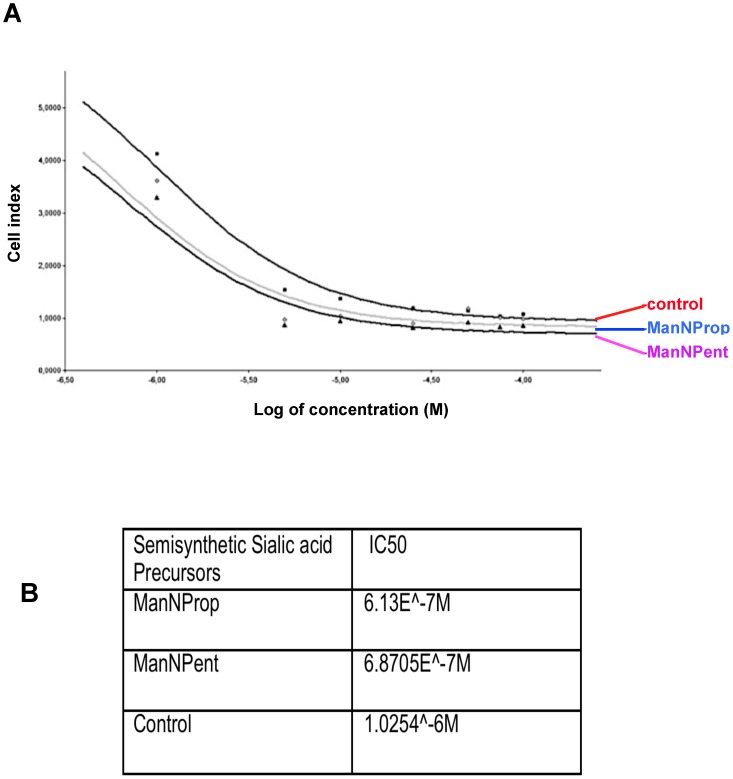
Cytotoxicity assay with 5-Fluorouracil. 0.05×10^6^ SHSY5Y cells were seeded in the RTCA E plate and cultured in the presence or absence of 10 mM ManNProp or ManNPent followed by treatment with 5-FU. A. Cytotoxicity induced by 5-FU towards the engineered cells was measured. B. ManNProp and ManNPent treated cells displayed significant increase in cytotoxicity at 10-fold reduced 5-Fluorouracil concentration compared to untreated cells. ** P<0.0001 for ManNProp and ManNPent treatment until 25 µM 5-Flurouracil.

**Figure 6:**
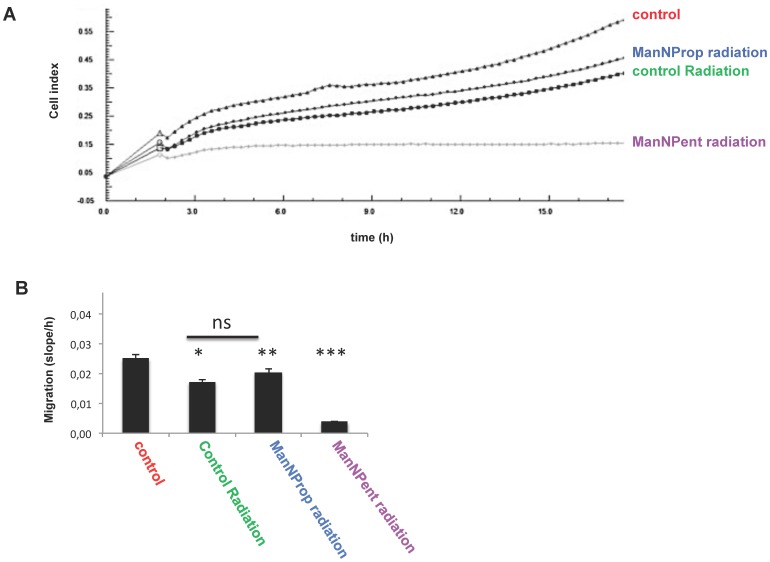
Radiation treatment impact on migration. SHSY5Y cells were cultured in the presence or absence of 10 mM ManNAc, ManNProp or ManNPent. Cells were radiated at a dose rate of 2 Gray/min for the total of 6 Gray. Radiated and control cells were subjected to migration assay (RTCA). (B) ManNProp and ManNPent pretreated cells displayed diminished migration ability at a significant rate (**P<0.0004, ***P<0.0001). Radiation treated control cells display reduced migration but not as significant (p<0.05) as compared to Sia engineered cells, ns: not significant.

The cell surface hydrophobicity has a significant impact on the uptake and effectiveness of chemotherapeutics. Therefore we analyzed the activity of two selected chemotherapeutics (5-fluorouracil and cisplatin) on SH-SY5Y neuroblastoma cells. First we calculated the IC50 values of 5-FU using a real-time cell analyzer. Metabolic Sia engineering of the surfaces of SH-SY5Y cells using ManNProp or ManNPent increased the sensitivity towards 5-FU up to ten fold with no significant difference between ManNProp or ManNPent (Fig.5 A, B). The sensitivity towards cisplatin was quantified using a MTT based proliferation assay. Table 1 summarizes that ManNPent engineered cells display increased sensitivity towards the cisplatin drug at low doses compared to the untreated controls. Metabolic Sia engineering with Sia precursors, followed by radiation treatment, led to almost complete absence of migration under hypoxic condition, however no significance difference has been observed between untreated and ManNProp treated cells (Fig. 6 A, B).

**Table 1:** MTT Assay for Cisplatin Treatment on Sia Precursor Pretreated SHSY5Y Cells.

Cisplatin	Percentage of viable cells		
Concentration (µM)	ManNProp	ManNPent	Untreated
0.1	96	79	100
0.5	93	76	98
2	77	64	82
3	74	63	76
5	58	51	66

SHSY5Y cells were cultured in the presence of 10 mM ManNProp or ManNPent followed by cisplatin treatment for 48 h. Complete medium was supplemented with MTT reagent and the absorbance at 560 nm was measured. ManNPent pretreatment displayed significant cytotoxicity (P<0.0001 to P<0.0056) for 0.1 to 5 µM cisplatin concentration. ManNProp treatment does not yield a significant difference in comparison to control cells.

## Discussion

Sia play an important role in the metastasis of tumor cells and especially during migration. This is evidenced by studies on various types of cancer [6], [10]. We have demonstrated that Sia metabolic engineering with the non-natural Sia precursors ManNProp and ManNPent led to reduced expression of both Sia and polySia at the cell surface and in total. Sia containing oligosaccharides are synthesized by about 20 sialyltransferases [23], whereas polySia is synthesized by two specific enzymes ST8Sia2 and ST8Sia4 [12]. In tumor cells mostly ST8Sia2 is re-expressed or strongly up-regulated and correlates with the increase in tumor grade [24]-[27].

Cell-cell interaction is the initial step and foremost important process for the starting point for metastasis. Both, mono- and polySia, strongly modulate the function of the respective sialoglycoprotein and/or sialoglycolipid. Neuroblastoma tumors and certain cell lines express NCAM-associated polySia, which is involved in metastasis by decreasing cell adhesion and promoting invasion [24], [25]. Therefore, we assume high expression of polySia as a negative prognostic marker. Thus, NCAM-associated polySia is a suitable target for tumor characterization and therapy [25].

Previous studies from our group have demonstrated a reduced polysialylation through selective inhibiton of ST8SiaII through application of modified Sia precursors [28]–[29]. In order to develop a novel strategy interfering mono-and polysialylation of neuroblastoma glycoconjugates, we have chosen this metabolic Sia engineering technique. As evidenced by FACS and HPLC analysis both the mono- (Neu5Ac) as well as polySia (α-2,8 linked Neu5Ac) expression was strongly reduced after application of non-natural Sia precursors.

Interestingly, metabolic Sia engineering with non-natural sialic acid precursors reduces the migration and invasion ability of the SH-SY5Y neuroblastoma cells. These neuroblastoma cells express significant amounts of NCAM and polySia. We expect from our Sia analysis that NCAM associated non-natural polySia possibility hinders the migratory and adhesion properties of the cancer cells. Since the involved glycoproteins possibly carry non-natural Sia, they also possibly play a role in reduced functional properties of the involved cells. However, further investigations using polysialyltransferases (ST8SiaII, ST8Sia IV) knockdown or polySia negative cell lines, are required to confirm the effects mediated by inhibition of polysialic acid through MSE. This will further help in identifying the mechanism behind the reduced function as well as to study the Sia composition of the macromolecules.

We also observed an interesting phenomenon towards enhanced sensitivity on treatment with anticancer drugs. 5-Fluorouracil exhibited increased sensitivity after engineering with non-natural Sia precursors (ManNProp or ManNPent). Cisplatin treatment also displayed an increased sensitivity on low doses. Cells expressing non-natural Sia, which are more lipophilic, may inhibit the interaction and also possibly reduce the function of multidrug transporters and also enable effective entry of the anticancer drug. Furthermore, they may act on the cancer cells by inhibiting the pro-invasive oncogenes [31] or by interfering with cellular signaling cascades [30]. Analysis of the composition of natural and artificial sialic acids (total and polySia) on MSE treated cells, as well as the exact mechanism behind the increased sensitivity towards chemo and radiation-therapy are underway.

Our data suggest that metabolic Sia engineering can be used as sensitizers for chemo and radiation therapy, as shown here in the neuroblastoma cell line model SH-SY5Y, and potentially in other tumors [32], [33]. Further experiments in the additional cell lines and in the *in vivo* mouse model using peracetylated modified Sia precursors are necessary for the elucidation of the mechanism. This will help in rationalizing metabolic sialic acid engineering with modified peracetylated sialic acid precursors as an effective therapeutic strategy for cancer treatment.

## Supporting Information

Figure S1 **Metabolic engineering of Sia in the neuroblastoma cancer cells.** Modified Sia precursors enter the cells via a passive transport mechanism and are integrated into the sialic acid biosynthetic pathway. The bifunctional enzyme UDP-GlcNAc epimerase/ManNAc kinase converts the modified precursors into the modified phosphorylated ManN(-R)-6-phosphates, followed by the formation of Neu5N(-R)- phosphates, and dephosphorylation to produce modified Sia. These modified Sia are transferred to the various glycoproteins such as NCAM, integrins, receptor tyrosine kinases (RTKs) or growth factors. They reduce the concentration of native Sia by inhibiting the sialyltransferases.
(TIFF)
