# Correction: Sialic Acid Metabolic Engineering: A Potential Strategy for the Neuroblastoma Therapy

**DOI:** 10.1371/journal.pone.0154289

**Published:** 2016-04-19

**Authors:** Vinayaga S. Gnanapragassam, Kaya Bork, Christina E. Galuska, Sebastian P. Galuska, Dagobert Glanz, Manimozhi Nagasundaram, Matthias Bache, Dirk Vordermark, Guido Kohla, Christoph Kannicht, Roland Schauer, Rüdiger Horstkorte

The following information is missing from the Funding section: “This work was supported by a grant of the von Behring-Roentgen-Stiftung to SPG.”
