# Supplementary figures and images for: Sialic Acid Metabolic Engineering: A Potential Strategy for the Neuroblastoma Therapy
Source: PLoS One. 2014 Aug 22;9(8):e105403. doi: 10.1371/journal.pone.0105403 (PMC4141789; doi:10.1371/journal.pone.0105403)

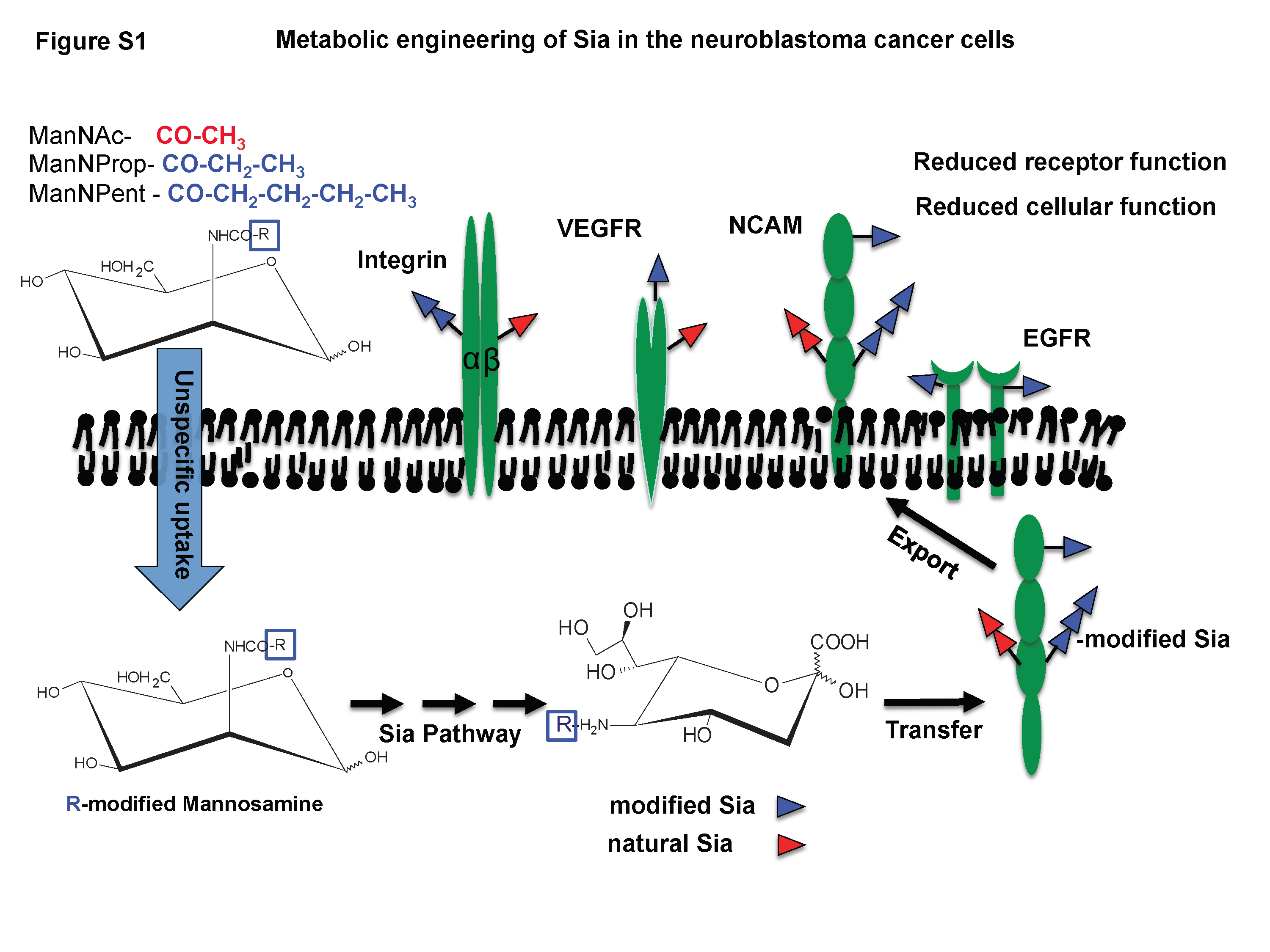

Supplement: Figure S1 — Metabolic engineering of Sia in the neuroblastoma cancer cells. Modified Sia precursors enter the cells via a passive transport mechanism and are integrated into the sialic acid biosynthetic pathway. The bifunctional enzyme UDP-GlcNAc epimerase/ManNAc kinase converts the modified precursors into the modified phosphorylated ManN(-R)-6-phosphates, followed by the formation of Neu5N(-R)- phosphates, and dephosphorylation to produce modified Sia. These modified Sia are transferred to the various glycoproteins such as NCAM, integrins, receptor tyrosine kinases (RTKs) or growth factors. They reduce the concentration of native Sia by inhibiting the sialyltransferases. (TIFF) [file pone.0105403.s001.tiff]
